# Fecal diagnostics in combination with serology: best test to establish STEC-HUS

**DOI:** 10.1007/s00467-016-3420-7

**Published:** 2016-05-30

**Authors:** Kioa L. Wijnsma, Sheila A. M. van Bommel, Thea van der Velden, Elena Volokhina, Michiel F. Schreuder, Lambertus P. van den Heuvel, Nicole C. A. J. van de Kar

**Affiliations:** 1 Department of Pediatric Nephrology, Radboudumc Amalia Children’s Hospital, Radboud University Medical Center, P.O. Box 1901, 6500 HB Nijmegen, Netherlands; 2 Department of Laboratory Medicine, Radboud University Medical Center, Nijmegen, Netherlands; 3 Department of Pediatrics, University Hospital Leuven, Leuven, Belgium

**Keywords:** Serological LPS antibody detection, STEC serotype O157, STEC-HUS, Anti-O157 antibodies, Diagnostics

## Abstract

**Background:**

In the majority of pediatric patients, the hemolytic–uremic syndrome (HUS) is caused by an infection with Shiga toxin-producing *Escherichia coli* (STEC), mostly serotype O157. It is important to discriminate between HUS caused by STEC and complement-mediated HUS (atypical HUS) due to differences in treatment and outcome. As STEC and its toxins can only be detected in the patient’s stool for a short period of time after disease onset, the infectious agent may go undetected using only fecal diagnostic tests. Serum antibodies to lipopolysaccharide (LPS) of STEC persist for several weeks and may therefore be of added value in the diagnosis of STEC.

**Methods:**

All patients with clinical STEC-HUS who were treated at Radboud University Medical Center between 1990 and 2014 were included in this retrospective single-center study. Clinical and diagnostic microbiological data were collected. Immunoglobulin M (IgM) antibodies against LPS of STEC serotype O157 were detected by a serological assay (ELISA).

**Results:**

Data from 65 patients weres available for analysis. Fecal diagnostic testing found evidence of an STEC infection in 34/63 patients (54 %). Serological evidence of STEC O157 was obtained in an additional 16 patients. This is an added value of 23 % (*p* < 0.0001) when the serological antibody assay is used in addition to standard fecal diagnostic tests to confirm the diagnosis STEC-HUS. This added value becomes especially apparent when the tests are performed more than 7 days after the initial manifestation of the gastrointestinal symptoms.

**Conclusions:**

The serological anti-O157 LPS assay clearly makes a positive contribution when used in combination with standard fecal diagnostic tests to diagnose STEC-HUS and should be incorporated in clinical practice.

## Introduction

Hemolytic–uremic syndrome (HUS) is diagnosed when the characteristics of hemolytic anemia, thrombocytopenia, and acute renal failure are present [1]. There are different etiologies leading to HUS, with the Shiga toxin-producing *Escherichia coli* (STEC) HUS and complement-mediated atypical HUS (aHUS) being the most prominent ones. In more than 90 % of cases, HUS follows a gastrointestinal infection with STEC. Additionally, over 50 % of STEC-HUS cases are due to STEC serotype O157, although other serotypes, such as O26, O103, O145, and O111, are increasingly associated with HUS as well [2–5]. With symptomatic treatment, patients with STEC-HUS often recover spontaneously, with only a small number progressing to end-stage renal disease (ESRD) [6]. In contrast, aHUS generally has a poor outcome, with 2–10 % mortality among patients in the acute phase of the disease and up to 50 % of patients progressing to ESRD [7].

It is therefore vital to be able distinguish between STEC-HUS and aHUS, as this has major consequences in terms of treatment possibilities (for example, the use of the expensive orphan drug eculizumab, which is currently the standard treatment for aHUS) and outcome [8]. However, it can be challenging to differentiate clinically between these two entities due to their similar symptoms. For example, in 6–10 % of children with STEC-HUS there is no (bloody) diarrhea, whereas aHUS is preceded by diarrhea in 25 % of the cases [7, 9]. As HUS is often diagnosed *per exclusionum*, providing proof for the presence of STEC forms the basis for differentiation between aHUS and STEC-HUS [2].

In most laboratories, an STEC infection can only be confirmed by an examination of fecal material, mostly through simultanous testing of stool culture, shiga toxins immunoassays, and/or polymerase chain reaction PCR assays for the detection of shiga toxin (Stx) genes [10]. With stool cultures, the presence of an STEC can only be established in a limited number of patients (approx. 30–69 %) with clinical signs and symptoms of HUS [4, 11]. The additional use of PCR for detection of Stx genes increases the odds of finding evidence of an STEC infection up to 70 % [12, 13]. However, the presence of STEC in the intestines declines rapidly during the first week of the illness [10, 14], whereas the average time between the first day of diarrhea and the development of HUS is 5–13 days [1, 2].

Over two decades ago, Chart et al. described the use of serological assays to detect antibodies against, among others, serotype O157 lipopolysaccharide (LPS) as a diagnostic tool to establish an STEC infection [15–18]. A few studies have subsequently shown that in patients with clinical signs and symptoms of HUS, for whom only a limited number of the stool cultures were positive, the results of serological testing for STEC were positive in 60–94 % of the patients [4, 11, 19]. The most important explanation for this low number of positive stool cultures next to the low inoculums of the bacteria is the small time window when STEC can be detected in the feces. In contrast, the antibody response, composed of immunoglobulin M (IgM), can be detected from 5 days up to 2 months after the onset of the symptoms [20, 21].

Based on these characteristics, a broad application of serological assays for STEC may be expected. Additionally, in most of the microbiological studies conducted to date, various diagnostic techniques have been compared using conventional and molecular methods; however, all of these methods depend on the presence of fecal material [13, 22]. Hence, only limited data have been published on the combined use of fecal diagnostic testing and the serological antibody assay in patients with STEC-HUS. A review of the literature and discussion with colleagues suggested to us that interest in the serological antibody assay for the detection of an STEC infection has been largely neglected in past years.

In th study reported here, we assessed the added value of the serological anti-O157 antibody assay in combination with fecal diagnostic testing in pediatric patients with a clinical STEC-HUS. We also examined the time window between the onset of symptoms and testing for STEC using fecal diagnostic tests and a serological antibody assay.

## Materials and methods

This was a retrospective single-center study which included all patients who presented with a clinical pattern of STEC-HUS between 1990 and 2014 to the Pediatric Nephrology department of the Radboud University Medical Center Amalia Children’s Hospital. All available clinical and diagnostic data were collected. The diagnostic data included the results of all fecal diagnostic tests [stool cultures, cell cytotoxicity assays [free fecal Stx test (FStx), used in our center until June 2011] and PCR analyses for Stx genes (from June 2011 onwards). An enzyme-linked immunosorbent assay (ELISA) was used as the serological assay for IgM antibodies against O157 LPS [15].

A clinical pattern of STEC-HUS was defined as signs of a thrombotic microangiopathy, hemoglobin level below the lower limit of normal for a specific age, signs indicative of hemolysis, acute renal failure, thrombocytopenia of <150 × 10^9^/l, and (bloody) diarrhea or family members with diarrhea. Clinical data at presentation and follow-up were collected from the medical records. Hypertension was defined as repeated blood pressure measurements above the 95th percentile for sex, height, and age [23]. Anuria was defined as a urine production of <0.1 ml/kg/h for at least 12 h, and oliguria was defined as a urine output of <0.5 ml/kg/h. Neurological involvement was indicated by apathy, irritability, reduced consciousness, seizures, paralysis, encephalopathy, and coma, and signs of pancreatic involvement included pancreatitis and diabetes mellitus. The estimated glomerular filtration rate (eGFR) was calculated with the Schwartz formula (k-coefficient = 36.5) [24]. Extent of renal impairment was based on guidelines from Kidney Disease: Improving Global Outcome [25]. The onset of disease was defined as the first day of diarrhea. The time window of the fecal diagnostic tests and serological antibody assay was defined as the time between the onset of the symptoms and the collection of feces and/or serum.

### Fecal diagnostic tests

Feces were collected as soon as possible after admission to the hospital. In cases where feces could not be obtained, a rectal swab was done. Fecal material was plated on Sorbitol MacConkey agar containing 1 % sorbitol and on blood agar. After 24 h of incubation, non-sorbitol fermenting colorless colonies were tested for agglutination with anti-O157 O-antigen serum. Until 2011, fecal samples were also tested for the presence of FStx using cell cytotoxicity assays as previously described by Karmali et al. [26, 27]. In June 2011, the PCR assay for the detection of* Stx1*,* Stx2* and virulence genes, *E. coli* attaching and effacing gene (*eae*), and enterohemorrhagic *E. coli* hemolysin (*hly*) in feces replaced the time-consuming cell cytotoxicity assays as a standard test, in addition to the stool cultures. Nowadays, when a patient is suspected of HUS the feces is first tested for the presence of STEC with a PCR assay. When the assay results are positive, indicating STEC infection, a stool culture on Sorbitol MacConkey agar plates is performed. To further determine all strains, we send all isolates to the Dutch National Institute for Public Health and Environment (RIVM).

### Anti-O157 LPS assay

Serum was obtained from all patients and stored at −80 °C until analysis. Sera were screened for antibodies against the LPS serotype O157 with an ELISA as previously described by Chart [15]. In brief, the ELISA plates were coated with LPS from *E. coli* O157:H7 [List Biological Laboratories Inc., Campbell, CA; product code 206, diluted in carbonate buffer (pH 9.6) to a concentration of 20 ug/ml]. After incubation overnight at 4 °C, the plates were blocked, and diluted serum was added to the plate together with predetermined positive and negative control sera. After addition of the antibody goat anti-human IgM (Sigma-Aldrich, St. Louis, MO; product code A0420, diluted 1/500 with phosphate buffered saline with bovine serum albumin) and substrate (*p*-nitrophenyl phosphate tablets in diethanolaminebuffer), the absorbance was measured at 405 nm. A positive IgM reaction was defined by a mean absorbance of >0.800; values of <0.400 were to be considered negative and values between 0.400 and 0.800 were considered to be dubious and as such taken to be negative for the purpose of this study.

### Statistics

All available clinical variables for each patient were included in the analysis. Clinical values were expressed as valid percentages for categorical variables and as the mean and standard deviation or median and 25–75 percentile (interquartile range) for continuous variables, as appropriate. Values for the serological assays, stool cultures, FStx, or PCR that were inconclusive or missing were classified as a negative test result. The Chi-square test was performed to compare categorical data and the two-sided* t* test or Mann–Whitney* U* test was used to compare continuous data. To compare positive and negative test results in relation to the time since onset of disease, we used binary logistic regression analyses, and dummies were computed for the categorical variables.* P* values of <0.05 were considered to be statistically significant.

## Results

### Patient characteristics

During the period between 1990 and 2014, 72 children with a clinical pattern of STEC-HUS were seen in the Radboud University Medical Center Amalia Children’s Hospital. Seven patients were excluded because no data on feces and serology were available for further analysis. The patient characteristics of the 65 children with a clinical pattern of STEC-HUS are described in Table 1. The majority of patients (79 %) presented with STEC-HUS before the age of 6 years. All but two children had diarrhea at presentation (97 %), which was generally bloody diarrhea (79 %). One patient died in the acute phase of the disease from a systemic inflammatory response syndrome combined with severe STEC-HUS, as proven by positive results for both the fecal diagnostic tests and the serological assay. Another patient, with proven STEC-HUS based on fecal and serological tests, did not show any recovery of renal function and subsequently was placed on hemodialysis before undergoing kidney transplantation.

**Table 1:** Patient characteristics of 65 pediatric patients with a clinical pattern of hemolytic–uremic syndrome mediated by Shiga toxin-producing* Escherichia coli*

Patient parameters^a^	All patients
Male	52 %
Age of onset (years)	2 (1–4)
0–24 months (*n* = 18)	17 (10–20)
2–6 years (*n* = 33)	3 (2–4)
≥6 years (*n* = 14)	11 (9–12)
Symptoms at presentation	
Fever	27 %
Diarrhea, total	97 %
Proportion of which was bloody	79 %
Oliguria	29 %
Duration (days) (*n* = 5)	5 (3–8)
Anuria	59 %
Duration (days) (*n* = 26)	8 (7–10)
Blood pressure	
<95 percentile for age and height	34 %
≥95 percentile for age and height	66 %
Neurological involvement	34 %
Pancreas involvement	3 %
Biochemical evaluation at presentation (reference range)	
Hemoglobin (6.0–9.0 mmol/l)	5.8 (4.8–6.7)
White blood cells (5.0–13.0 × 109/l)	15.3 (10.9–22.3)
Platelet count (210–430 × 109/l)	47 (30–73.5)
Haptoglobin (0.3–1.6 g/l)	<0.08 (0.05–0.08)
LDH (<250 U/l)	3720 (2325–5809)
eGFR (>90 ml/min.1.73 m^2^)	15 (10–24)
Treatment	
Dialysis	63 %
Duration of dialysis (days)	10 (7–15)
Erythrocytes transfusion	80 %
≥ transfusions	11 %
Follow up	
eGFR at discharge (ml/min.1.73 m2)	55 (43–66)
eGFR < 60 after 5 years follow up (n = 8) (ml/min.1.73 m^2^)	4 (6 %)
Received kidney transplantation	1 (2 %)
Diseased	1 (2 %)

Categorical values are presented as an absolute number and/or as a percentage of the total. Continuous variables are presented as the median with the interquartile range (IQR) in parenthesis

LDH, actate dehydrogenase; eGFR, estimated glomerular filtration rate

^a^The numbers of patients (*n*)  for whom data were available are reported in parentheses

### Serological assays in addition to fecal diagnostic tests

**Fig. 1:**
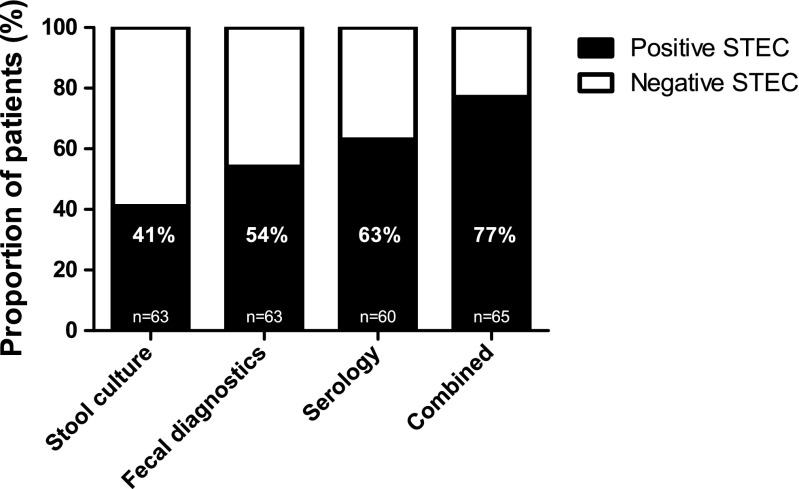
Serological anti-O157 antibody assay in addition to the standard fecal diagnostic tests. Test results from all 65 patients with a clinical pattern of hemolytic–uremic syndrome mediated by Shiga toxin (Stx)–producing* Escherichia coli* (*STEC*-HUS) for whom serology and fecal diagnostic tests were performed to confirm a STEC infection. When the fecal diagnostic test results (stool culture, cell cytotoxicity assay, and/or PCR) are combined with those of the serological antibody assay, 77 % of the patients had a confirmed STEC infection (*p* < 0.0001)

Evidence of an STEC infection was found in 50 patients (77 %) of the 65 patients with a clinical pattern of STEC-HUS. Fecal diagnostic tests identified STEC in 34/63 patients (54 %); in 26 of these 34 patients a STEC strain could be isolated with culture. Serological evidence of an STEC O157 infection was found in an additional 16 patients, which is an added value of 23 % (*p* < 0.0001) when fecal diagnostic tests are combined with the serological antibody assay to confirm the diagnosis of STEC-HUS (Fig. 1; Table 2). Among those patients with negative stool cultures, the PCR assay was positive for STEC-HUS in three patients and the cell cytotoxicity assay (FStx) was positive for STEC-HUS in five patients. Since implementation of the PCR assay in 2011, Stx genes have been detected in nine (69 %) of the 13 patients who presented with HUS. Of these nine STEC infections, seven were also detected by serological assays. Three additional patients who tested negative for Stx genes in repeated PCR assays showed serological evidence of an STEC infection (Table 2).

**Table 2:** Proportion of positive results for the serological antibody assay for serotype O157 in relation to the fecal diagnostic test results

Diagnostic tests	Positive results for the serological assay	Negative results for the serological assay	Total (%)
Serology data	38 (63 %)	22 (37 %)	60
Missing			5
Fecal diagnostics			63
Missing serology			4 (6 %)
Positive	22 (65 %)	12 (35 %)	34 (54 %)
Negative	15 (60 %)	10 (40 %)	25 (40 %)
Stool culture			63
Missing serology			4 (6 %)
Positive	18 (69 %)	8 (31 %)	26 (41 %)
Negative	19 (58 %)	14 (42 %)	33 (53 %)
FStx, until 2011			33
Missing serology			1 (3 %)
Positive	10 (59 %)	7 (41 %)	17 (52 %)
Negative	9 (60 %)	6 (40 %)	15 (45 %)
PCR, since 2011			13
Missing serology			–
Positive	7 (78 %)	2 (22 %)	9 (69 %)
Negative	3 (75 %)	1 (25 %)	4 (31 %)

Test results from all 65 patients with a clinical pattern of Shiga toxin-producing *Escherichia coli*-hemolytic uremic syndrome (STEC-HUS) where the serological assay and fecal diagnostic tests were performed to confirm a STEC infection

Data in table are presented as the absolute number of patients with the percentage of patients a positive or negative serology result, respectively, given in parentheses. In a few patients, only fecal diagnostic tests were performed; the missing serology values are presented in the table

FStx, Free fecal shiga toxin test

Two addtional serotypes, serotypes O26 (*n* = 3) and O5, were detected in fecal specimens from three and one patient, respectively,sent to the RIVM for further testing.

### Time window between onset of disease and sample collection

The median time between onset of disease and the collection of feces and serum was 7 (IQR 5–9) and 8 (IQR 5–12) days, respectively. The added value of serology became more evident when the fecal and serological diagnostic tests were run on specimens collected ≥7 days after the start of the symptoms (Table 3). Within 7 days after the first manifestation of the symptoms, 14 patients had negative fecal diagnostic test results; of these, five (36 %) had positive results on the serological assay, confirming a STEC infection. However, of the 12 patients with negative fecal diagnostic test results based on fecal specimens collected ≥7 days after the start of the disease, eight (67 %) were found to test positive for O157 on the serological assay. Hence, when feces and serology specimens were collected ≥7 days after the start of the symptoms, the serological antibody assay had an added value of 33 %, which is significantly higher than the 14 % added value when the specimens for testing were collected within 7 days (*p* = 0.024).

**Table 3:** Added value of serology in relation to the time window between onset of symptoms and collecting material

Diagnostics	Proven STEC infection		
	Total	<7 days	≥7 days
Fecal diagnostics	54 % (34/63)	44 % (11/25)	62 % (20/32)
Serology	62 % (38/61)	45 % (9/20)	69 % (25/36)
Combined: fecal diagnostics & serology	77 % (50/65)	58 % (7/12)	95 % (20/21)
Added value of serology	23 %	14 %	33 %

Collection of diagnostics in the first 6 days after the start of the symptoms are compared with 7 days or more since onset and the influence on the detection of Shiga toxin-producing Escherichia coli (STEC) infection is compared. The added value of serology becomes more evident as of 7 days (*p* = 0.024)

## Discussion

Various fecal diagnostic tests are recommended in the literature to establish an STEC infection as the cause of HUS [10], including stool cultures, Stx immunoassays, cell cytotoxicity assays, and PCR for Stx genes. However, limited data are available published on the benefits of combining fecal diagnostic with serodiagnostic testing in patients with STEC-HUS. The results of our retrospective study show that the serological anti-O157 antibody assay is of additional value to the fecal diagnostic tests used in patients with a clinical STEC-HUS. In our study, use of the standard fecal diagnostic tests resulted in a STEC infection being detected in half of the HUS patients; in comparison, evidence of an STEC infection was detected in 77 % of the 65 patients when serological testing was used in combination with the standard fecal diagnostic tests. The added value of serological testing further improves when the specimens for testing were collected ≥7 days after the start of the symptoms.

Our results are in line with those reported by Espié et al. [4] who studied 900 children with HUS and tested for STEC by stool culture, PCR assay for Stx genes, and serological assays for multiple serotypes. In 232 (37 %) children, The presence of a STEC was confirmed in 232 (37 %) children with a stool culture and in 37 patients Stx was detected with the PCR assay. STEC infection was confirmed in 518 (60 %) patients based on positive serological results, of which 85 % of patients had serotype O157. The combined use of fecal diagnostics and serology provided evidence of a STEC infection in 590 (66 %) patients. Unfortunately, no further information on the time window was provided by the authors.

The fecal diagnostic tests (stool culture, cell cytotoxicity assay, and/or PCR) detected STEC in only half of our patients with a clinical pattern of STEC-HUS. A few factors may explain this low number of positive stool cultures, all of which are linked to the time of presenting in relation to the development of the disease. First, the isolation rate of STEC in feces declines quickly after the first manifestation of the gastrointestinal symptoms, and most parents seek medical attention after the first signs of bloody diarrhea (which is after approx. 3 days). Secondly, there is a low inoculum whereby the odds of finding a STEC in the feces is also limited during the first 7 days. The third and final factor is that the diarrheal prodrome has often ceased before the onset of HUS, making detection of the pathogen or its toxins rather difficult when the fecal speciment is collected at the time of presenting with a HUS [1]. Frequent stool collections may be considered one option to increase the chance to detect a STEC infection. Furthermore, new and promising techniques have been developed for the detection of STEC infection since the start of this retrospective study, as illustrated by the PCR assay for Stx genes, but also more recently by the molecular approach to assess the virulence profile and serotyping of STEC strains. In comparison to the other standard fecal diagnostic tests (e.g., stool culture and PCR), molecular testing is generally only performed in reference laboratories, as this approach is mostly used for epidemiological analysis [10, 28]. The PCR assay for Stx genes was introduced only a few years ago in our hospital, and data on this assay were only available for a minority of patients in our study, although results are promising. Even though these techniques are more precise in terms of detecting the presence of STEC, they still require fecal material for testing. Therefore, in cases without stool production, besides a rectal swab that is highly recommended, it may still be difficult to detect STEC.

In 15 (23 %) of the 65 patients enrolled in our study, the results of the serological assay and the fecal diagnostic tests were negative for a STEC infection. One explanation may be that the techniques used were not sufficiently sensitive, especially because the majority of the specimens were tested before the implementation of the PCR assay. Another possible explanation is the timing of sampling, even though the time between the onset of symptoms and the collection of fecal specimens in our cohort is comparable with those of other studies [4, 29]. In our study, only a serological antibody response against O157 was tested. O157 currently accounts for 30–80 % of STEC-HUS cases in The Netherlands, and our serological assay could not detect other important serotypes [4, 30, 31]. Also, the serum samples may have been collected too early in the disease course—that is, prior to seroconversion. When these explanations appear to be unlikely, other causes of HUS, such as aHUS, should be taken into account. However, during follow up, none of the 15 patients with negative fecal diagnostic and serological test results presented with a relapse indicative of aHUS or showed signs of permanent complement dysregulation.

Our results clearly show that a patient with HUS in the absence of positive fecal diagnostic test results is not equivalent to one with aHUS. Moreover, it may very well be a STEC infection that is causing the HUS despite the negative fecal diagnostic test results. The need for regular serological antibody assays in patients with HUS becomes even more important with the introduction of the new and very expensive complement inhibitor eculizumab as a treatment for aHUS. We recommend that this assay be performed centrally in specialized laboratories per country to guarantee the quality and reliability of the assay and thereby ensure its feasibility and affordability in general clinical use.

The added value of the serological assay in relation to the time window has not been studied previously. Our results show that this added value becomes more evident (14 vs. 33 % before and after 7 days, respectively) when the specimens are collected >7 days after the first manifestation of symptoms. The number of patients in Table 3 is quite small due to missing data on the time window. In addition, feces were collected within 7 days in 11 patients, whereas the serum was collected >7 days after the start of the symptoms, presumably due to negative fecal diagnostic test results. We anticipated the rise in positive serological assay results after 7 days, as IgM is detectable approximately 5 days after the onset of the symptoms [21]. In contrast, the rise in positive fecal diagnostic test results after 7 days was not expected considering the awaited rapid decrease of STEC in the feces. The high percentage of positive results for the fecal diagnostic tests is partly explained by the inclusion of the cell cytotoxicity tests (FStx) and PCR assays, both of which are highly sensitive tests, even after 7 days, as compared to stool culture techniques [20]. However, these techniques all rely on the availability of fecal material. We recommend testing for the presence of STEC with fecal diagnostic tests and serological test concurrently in HUS patients at admission because the time course of the disease at presentation is often unclear. In the case of negative fecal diagnostic and serological test results for specimens tested within 7 days after the start of the symptoms, our advice would be to retest for antibodies after ≥7 days since the onset of disease.

Based on our results, the serological antibody assay seems to be indispensable to establish a diagnosis of STEC-HUS; however, the role of serological antibody detection in healthy controls remains unclear and needs further exploration. In one study, 22 of the 606 tested healthy controls with high exposure to cattle carrying STEC had positive serological test results for O157. However the antibodies found were mainly IgG, which could be indicative of repeated exposure. Presumably, the IgM antibody response we test in this study is more indicative of an acute infection [32]. There is one important limitation of the serological anti-O157 antibody assay—namely, the possibility of cross-reaction with subsequent false negative results. Cross-reaction has been reported for pathogens such as as *Brucella abortus*, *Yersinia enterocolitica*,* Vibrio cholera*,* Escherichia hermanni*,* Citrobacter freundii*,* Citrobacter sedlakii*, and *Salmonella* [17]. However, these pathogens are rarely associated with the onset of HUS.

One limitation of our study is its retrospective nature. All information had to be gathered from medical records, thereby increasing the odds that the information would be difficult to interpret or even be missing. Furthermore, serological data from the antibody assay were available only on serotype O157; although serotype O157 is still the main serotype associated with HUS in the Netherlands, other STEC serotypes associated with HUS are being increasingly detected. However, it was not the purpose of this study to investigate the epidemiology of STEC, rather to evaluate the added value of the serological assay, which has been perform in our hospital since 1990, in addition to the fecal diagnostic tests to determine a STEC. STEC serotype O157 is still the cause of HUS in over 50 % of the cases in the Netherlands. The remaining serotypes are more scattered; for example, the second most prevalent serotype associated with HUS, serotype O26, accounts for <20 % of the HUS cases [4, 30, 31]. In our cohort, four patients had a confirmed STEC infection with a non-O157 serotype (tested by RIVM)—serotypes O26 and O5. To increase the detection of the pathogens causing HUS, we are currently updating the serological antibody assay for other important serotypes as well.

In conclusion, the serological O157 antibody assay is an important additional test for the confirmation of STEC, especially when the fecal diagnostic test results are not sufficient to establish a STEC infection. Moreover, it is essential to take the time window into account: when patients present ≥7 days after the start of the symptoms, the serological antibody assay could be indispensable to establish a diagnosis of STEC-HUS. We recommend the implementation of the serological antibody assay as standard diagnostic method in combination with the fecal diagnostic tests for all patients who present with a clinical STEC-HUS.
